# Genome Sequence of *Porphyromonas gingivalis* Strain HG66 (DSM 28984)

**DOI:** 10.1128/genomeA.00947-14

**Published:** 2014-09-25

**Authors:** Huma Siddiqui, Deborah Ruth Yoder-Himes, Danuta Mizgalska, Ky-Anh Nguyen, Jan Potempa, Ingar Olsen

**Affiliations:** a Department of Oral Biology, Faculty of Dentistry, University of Oslo, Oslo, Norway; b Department of Biology, University of Louisville, Louisville, Kentucky, USA; c Department of Microbiology, Faculty of Biochemistry, Biophysics and Biotechnology, Jagiellonian University, Krakow, Poland; d Institute of Dental Research, Westmead Centre for Oral Health and Westmead Millennium Institute, Sydney, Australia; e Department of Oral Biology, Faculty of Dentistry, University of Sydney, Sydney, Australia; f Department of Oral Immunology and Infectious Disease, School of Dentistry, University of Louisville, Louisville, Kentucky, USA

## Abstract

*Porphyromonas gingivalis* is considered a major etiologic agent in adult periodontitis. Gingipains are among its most important virulence factors, but their release is unique in strain HG66. We present the genome sequence of HG66 with a single contig of 2,441,680 bp and a G+C content of 48.1%.

## GENOME ANNOUNCEMENT

The Gram-negative anaerobic rod *Porphyromonas gingivalis* is one of the most important pathogens in chronic adult periodontitis (1), and is also thought to be related to systemic diseases such as cardiovascular diseases and rheumatoid arthritis (2, 3). Strains of *P. gingivalis* differ in pathogenicity (4). The major and primary virulence factors of *P. gingivalis* are gingipains (5). Strain HG66 is exceptional because it does not retain gingipains on the cell surface but releases the majority of proteases in a soluble form. Accordingly, HG66 secretes all carboxy terminal domain-bearing proteins as soluble substances (6) while other *P. gingivalis* strains glycosylate the same proteins and retain them on the cell surface. The genome sequence of HG66 may enable a better understanding of the protein secretion/glycosylation system of *P. gingivalis*. Complete genome sequences of strains ATCC 33277^T^, W83, TDC60, and SJD2 are already available (7–10). The aim of the present study is to present the full genome sequence of HG66.

HG66 (DSM 28984) was isolated in Roland R. Arnold’s laboratory at Emory School of Dentistry, Atlanta, GA and maintained in Jan Potempa’s laboratory since 1989. Prereduced, enriched trypticase soy broth (eTSB) was used as the growth medium. Genomic DNA was extracted using the Qiagen QIAamp DNA minikit and eluted in dH_2_O. The genome sequence was obtained by applying Pacific Biosciences RS technology (Pacific Biosciences, Menlo Park, CA). A 10-kb insert library using P4-C2 chemistry was prepared and sequenced on four single-molecule real-time (SMRT) cells. An average read length of 5,338 bp with ~200-fold coverage of the genome was obtained.

The HGAP protocol implemented by SMRT analysis version 2.0.1 was used to assemble the HG66 genome. The genome was annotated using NCBI Prokaryotic Genomes Automatic Annotation Pipeline (PGAAP) and RNAmmer (11). Additionally, the genome was analyzed on the Rapid Annotation using Subsystems Technology (RAST) server (12).

The genome of HG66 included a single contig with 2,441,680 bp and a G+C content of 48.1%. A total of 2,062 genes were annotated which comprised of 1,958 predicted coding sequences (CDSs), 53 tRNAs, and 12 rRNAs.

Annotation by RAST revealed 273 subsystems (sets of related functional roles) in the genome. The protein metabolism accounted for 205 subsystem feature counts including genes in the protein biosynthesis machinery, such as 34 large subunits and 23 small subunits of the bacterial ribosome, and 15 universal GTPases and tRNAs. Further, 151 cofactors, vitamins, prosthetic groups and pigments, 98 RNA metabolism, 97 DNA metabolism, and 81 carbohydrates subsystem features were observed. Membrane transport and protein metabolism showed high counts. This is interesting since protein secretion/glycosylation is unique in HG66. The modification and motif analysis report by the PacBio RS sequencer indicated that only adenine bases were methylated.

The availability of genome sequence of HG66 may offer the opportunity to better understand the protein secretion/glycosylation system of *P. gingivalis*.

### Nucleotide sequence accession numbers.

This genome sequencing project was deposited in GenBank, under accession no. CP007756 (*P. gingivalis* strain HG66). The version described is the first version.
